# On the development of a professional mandate by social workers in medical rehabilitation– key results from the SWIMMER Project

**DOI:** 10.3389/fresc.2024.1383995

**Published:** 2024-08-30

**Authors:** Tobias Knoop, Nadja Freymüller, Stephan Dettmers, Thorsten Meyer-Feil

**Affiliations:** ^1^ Faculty of Medicine, Institute for Rehabilitation Medicine, Interdisciplinary Centre of Health Sciences, Martin Luther University Halle-Wittenberg, Halle (Saale), Germany; ^2^ Endowed Professorship Rehabilitation Science | Health Services Research in Rehabilitation, School of Public Health, Bielefeld University, Bielefeld, Germany; ^3^ Institute for Social Work in the Life Course, OST-Ostschweizer Fachhochschule, St. Gallen, Switzerland

**Keywords:** goal orientation, professionalism, health services research, ethnography, social law, interprofessional collaboration

## Abstract

Social work in the German rehabilitation sector is practiced with great variation and its interventions lack research evidence. The SWIMMER project aims to develop a program theory of social work in rehabilitation to explain this variation and to discuss possible conditions. The dealing with ethical dilemmas by social workers is one possible influence and the focus of this paper. The social workers’ practice was analyzed using the triple mandate, a German-Swiss concept that describes three possible, sometimes simultaneous directives without a concrete call to action from society, the client or the profession. This qualitative, case-comparative research project collected data from interviews with social workers and managers, participant observation and counseling sessions in ten German rehabilitation facilities. Social workers were confronted with all three mandates. They prioritized either the societal mandate or the client mandate. A consequence for social work practice was the limitation of options under social law (mandate by society). Social workers relied on their professional experience to reflect on the mandates. They used a variety of strategies when faced with conflicting mandates. The research project has succeeded in systematizing the orientations of social workers in goal conflicts. Further investigation on this topic on a broader basis would be beneficial.

## 1 Introduction

Social work is a common service in rehabilitation, as the authors showed in recent reviews (1, 2). However, a review of German research demonstrated substantial variation in utilization rates and duration between patient groups and rehabilitation facilities (1). The latest available data show a wide distribution, with more than 90 percent of all German rehabilitants receiving at least one social work service during their stay (3). In addition to exceeding the recommended utilization rates of the German Pension Insurance Funds [in German: Deutsche Rentenversicherung (DRV)] (3). This wide distribution may not mean an effective allocation of social work resources. Unfortunately, there is little evidence on the effectiveness of social work interventions in German rehabilitation settings and the transferability of international research is unclear (1, 2). There is also a lack of evidence to guide resource allocation or to explain the wide variation of service provision (4, 5). In recent years, three German trials have been published that investigated the effectiveness of social work in rehabilitation. One trial reported a positive effect (6), another one displayed a negative effect (7), and a third trial showed no treatment effect at all (8). Due to the heterogeneity of the results, no consistent conclusion about the effectiveness of social work in the German setting can be drawn. Additionally, insufficient information was provided about the theoretical foundation of the investigated interventions. The DRV addressed these shortcomings and established practice guidelines for social workers in rehabilitation with a best-practice approach (9). The research project, “causal assumptions about social work in medical rehabilitation” [In German: Sozialarbeiterische Wirkmechanismen in der medizinischen Rehabilitation (The SWIMMER-Project)], takes a more fundamental approach. The aim was a theory-based evaluation of social work practice in the German rehabilitation setting (10). A resulting program theory should explain variation in practice and describe underlying mechanisms. The research project was designed with the intention of addressing goal conflicts, given the hypothesis that dealing with and resolving goal conflicts would be critical for social work practice in rehabilitation (11). The results of this endeavor are presented here. A brief description of the program theory that we have developed as a result of this study has been published elsewhere (12).

### 1.1 Medical rehabilitation in Germany and goal conflicts in vocational participation (study setting)

Medical rehabilitation in Germany is part of the German health system and corresponds to the international rehabilitation definition by Negrini et al. (13). Especially when return to work is possible, the DRV pays for the rehabilitation services. It provides the majority of medical rehabilitation services (approximately one million medical rehabilitation services in 2022) in Germany (3). The majority of these services are inpatient services (2022: 80%) and have an average duration of approximately 29 days for women (28 days for men) (3). An interprofessional team under medical supervision provides rehabilitation services. Namely, the professions of this team comprise medicine, nursing, occupational therapy, physiotherapy, psychology, exercise therapy and others (3). While there is no legal obligation to offer social work services to institutions providing the rehabilitation services, the DRV has developed several standards that include social work in rehabilitation practice (14). Aligning with the United Natiońs convention on the rights of persons with disabilities (CRPD), the German Social Code defines the global goal of all rehabilitation services as fostering autonomy and equal participation in society of disabled persons or persons at risk of being disabled. The implicit goal of all services paid by the DRV is the restoration of the vocational capacities of their insured persons (vocational participation) and the prevention (or delay) of disability pension benefits. A key instrument by the DRV to reach this goal is the evaluation of the rehabilitant's work-(dis)ability (15). This evaluation is mandatory for all rehabilitation facilities and rehabilitation physicians in all cases and is an important part of the discharge summary. It is a requirement that physicians working in rehabilitation have undergone specialized training in the evaluation of work-related disability. This two-dimensional evaluation assesses the patients’ work (dis)ability for his/her last job and any other job. Wind et al. (16) noticed similarities of the evaluation processes in the international setting. The evaluation of the rehabilitation physician provides access to certain social benefits. Again, in accordance with the CRPD, the potential recipients have a legally guaranteed right for self-determination and are free to choose the service provision and delivery (in German: “Wunsch- und Wahlrecht”). In this context, mutual agreement on rehabilitation goals (goal-oriented rehabilitation) is another expression of the patient´s right of self-determination (17). Rehabilitants do not always agree with the evaluation of work-related disability. Nagl and Farin (18) observed for the German rehabilitation system a discrepancy in vocational goals in 69% of rehabilitation physician-rehabilitant dyads. For example, patients were more likely to anticipate a return to work than physicians, given their respective illnesses or disability. Levack and colleagues (19) present a more comprehensive overview of the potential discrepancies that can arise, particularly when the patient's objectives extend beyond the scope of the vocational realm. These discrepancies may present a significant challenge for professionals and might be understood from an ethical perspective. These challenges are the second most important ethical issue for rehabilitation professionals, subsequent to issues relating to reimbursements and patients’ refusal to follow recommendations (20). As goal setting is considered to be a central factor in the effectiveness of rehabilitation interventions (21), the way of dealing with discrepant goals may have a detrimental effect on the efficacy of rehabilitation interventions.

### 1.2 Theoretical orientation: the triple mandate by staub-bernasconi

The theoretical background of the SWIMMER-Project emerged from two distinct disciplinary perspectives (11). On the one hand, the participating researchers have scientific backgrounds in public health, psychology and rehabilitation sciences (first author, second author and senior author). On the other hand, some of the researchers involved also have degrees in social work as well as practice experience as health social workers, thereby representing the social work perspective in the research team (first author, third author). This had an influence on the theoretical orientation of the SWIMMER-project. One of the referenced social work theories stems from Swiss social worker Silvia Staub-Bernasconi (22, 23). Staub-Bernasconi's social work theory involved the conceptualization of the so-called triple mandate. She defined mandates as directives without a concrete call to action and stated that social workers face mandates from three different perspectives. This theory assigns ethical obligations to key stakeholders and serves as a descriptive ethical framework in the event of conflicts of interest, such as goal conflicts (23).

The first mandate is the “societal mandate”, which encompasses two dimensions: help and control. On the one hand, the society—especially in well-established welfare systems—provides services to help those in need. Social work is sometimes part of these services or supports in applying for these benefits. In the German welfare system, the benefits for disabled or likely to be disabled individuals include public and non-public counseling services, medical and vocational rehabilitation, community rehabilitation interventions and disability pensions or other measures of financial security. Additionally, social work vets the benefit receipt. The control mandate is codified in law and expressed in the ubiquitous motto “Rehabilitation prior to retirement”. The control dimension of the societal mandate is often forwarded to the social workers. The second mandate is related to the clients of social work services. Clients may have explicit goals and communicate concerns, which can be understood as a mandate to the social worker to act on. However, not all clients are capable of “formulating a mandate”. In such cases, Staub-Bernasconi (19) states that it is the professional obligation of a social worker to identify and help to articulate the client's mandate. To challenge the demands of the two mandates, Staub-Bernasconi (19) calls for a third mandate, the professional mandate, which involves professional ethical standards, such as those proposed in medical ethics and rehabilitation. This two-fold mandate should be informed by evidence, too. As social work is a profession based on human rights and a people-first approach, social workers should prioritize the needs of their clients in the case of potential goal conflicts. The third mandate should advise the decision-making process, with the aim of mediating or resolving potential conflicts between the societal and client mandates. The existence of differing mandates can give rise to a number of potential issues, including the possibility of conflicts of loyalty and the emergence of goal conflicts. As vocational participation of the patient is a designated goal for social workers in rehabilitation (1), they are at the forefront of goal conflicts in vocational participation.

### 1.3 Ethical issues in social work and rehabilitation research

Social work as well as rehabilitation research address the triple mandate frequently under another heading. Shared decision-making and related dilemmas are subject of many research projects. One conflict of decision-making identified by Banks et al. (24) early in the COVID-19 pandemic from a short ethic report by health social workers was the decision whether to follow national and organizational policies or to use professional discretion. Ylvisaker and Rugkasa (25) described and analyzed conditions that can cause ethical dilemmas. They pointed out that ideological rules and discourses by the society as well as organizational conditions and conflicting loyalties impact social work practice and the understanding of social problems. A research project with social workers in Finland showed that conflicts in social work rehabilitation practice are primarily resolved through the application of the people-first principle (26).

In the field of rehabilitation research, Sabella et al. (27) discuss the formulation of a professional mandate through a recourse to so-called professional dispositions in rehabilitation counselors. McGlinchey & Davenport (28) analyze the decision-making of physiotherapists and identify multiple patient, therapist and organizational criteria as considerable factors. The concurrence of two mandates, here by the client and the society, is also subject of the street-level bureaucracy research. Maynard-Moody & Musheno (29) developed the “citizen agent” and “state agent” as two professional types of, for example, rehabilitation counselors. In a survey of rehabilitation professionals with open-ended questions, three themes of ethical issues emerged from the data. The professionals provided narratives that addressed questions pertaining to institutional ethics (e.g., healthcare reimbursement), professional practice (e.g., professionalism), or clinical decision-making (e.g., goal conflicts) (20).

This research relates to the three mandates and underlines their importance for social work and rehabilitation practice. The advantages of the concept we have applied in our research remain the integration of research results as a guiding orientation, which has been underrepresented in social work to date; the categorization of the multiple constraints social workers face and the assignment of these to important stakeholders. As the setting of rehabilitation goals is influenced by different interests, which my lead to conflicting goals (17), the triple mandate is an appropriate approach for describing the ethical dilemmas that may result from these conflicting goals. The triple mandate is a well-known concept in social work theory, but there is a lack of empirical research focusing specifically on the three mandates. Its application in rehabilitation is novel. Consequently, we sought to gain empirical insights into the triple mandate and to use this framework in order to analyze how social workers in rehabilitation address and resolve goal conflicts. We postulated that this action and the mediation of different mandates have an impact on social work practice in rehabilitation. This paper reports on the extent to which Staub-Bernasconi's theory applies to social work practice in rehabilitation and the contribution this approach can make in explaining social work practice variation.

## 2 Methods

In this qualitative research project with a case-comparative approach (30, 31), the researchers collected interview data and data from participant observations in two phases of data collection at ten rehabilitation facilities in the federal state North Rhine-Westphalia, Germany (the largest federal state within Germany with a population of approximately 18 million). The research project has been reviewed and approved by the ethics committee of Bielefeld University (No. 2021–23). All participants in the interviews and recorded counseling sessions provided written informed consent.

### 2.1 Sampling

For the first phase from 09/2020 to 04/2021, a maximum variation sampling technique was applied. Sampling criteria mainly referred to the rehabilitation facilities itself and were, for instance, the primary diagnostic group and service type (inpatient vs. outpatient) (32). The facilities for the second phase from 10/2021 to 02/2022 were selected based on a theoretical sampling approach (33). The development of sampling criteria was informed by the initial analysis of the data from the first six participating rehabilitation facilities. The developed sampling criteria relevant to this paper were, for instance, the presence of an intraprofessional supervisor for the social service, employees affiliated with the social service with no social work background, and social services conducting regular team meetings. The researchers recruited the rehabilitation facilities through personal contacts and with support from the funders (non- and for-profit service providers) of the endowed professorship, the research project was affiliated with, and the German Association of Health Social Workers [in German: Deutsche Vereinigung für Soziale Arbeit im Gesundheitswesen (DVSG)]. In both sampling strategies, the researchers contacted the leading staff or the social workers directly.

### 2.2 Data collection

Data were collected via semi-structured interviews with social workers and leading staff members, and participant observations of the social workerś practice with anonymous ex-post protocols, written by the researchers. Additionally, social workers recorded their own counseling sessions when applicable. The first and second authors collected all data in a three-day visit in each rehabilitation facility. In the interviews, the social workers were given the opportunity to elaborate on their practice. An interview guide was developed based on sensitizing *a priori* concepts and used during the interview process (Table 1). In the initial thematic block, the participants were prompted to elucidate their routine occupational practices, or their interactions with social service agencies, in everyday settings. Follow-up questions were posed to elicit further information related to the provided narratives, as outlined in Table 1. In particular, the questions addressed issues related to interprofessional collaboration, case studies illustrating successful outcomes, and supporting and limiting working conditions. The ex-post protocols of the participant observations were recorded chronologically.

**Table 1:** Central topics of the interviews.

Interviews with social workers	Interviews with leading staff
Everyday practice	Social work in the rehabilitation process
Interaction with rehabilitants	Social work and the interprofessional team
Collaboration with the interprofessional team and external actors	Success
Success	Competencies
Competencies	Contextual factors
Contextual factors	

### 2.3 Data analysis

All recorded data were transcribed in German according to the rules of Dresing and Pehl (34). To protect the confidentiality of the respondents, in the report of the data the gender of the participants was partially reversed, with males assigned to the female category and vice versa. In order to describe the mandates as important phenomena and to link them to the (inter)/action of relevant actors, the data analysis was guided by a grounded theory (GT) approach (35, 33). The first and second authors started the analysis with data from one rehabilitation facility. Initially, they read all transcripts and protocols. To structure the great amount of data, the research team started to develop some codes inductively with a focus on frequently recurring themes observed in the practice of social workers. Additionally, they employed deductive coding, referencing the triple mandate or the International Classification of Functioning, Disability and Health (ICF), for instance. Subsequently, they analyzed the data in a more reconstructive manner by considering some strategies made to theorize the data (35, 33). More specifically, the analysis pursued an approach that constantly compares cases to show features that those cases have or do not have in common. New cases could be either another social worker in the same team or different social services and their social workers from the other facilities. Another approach that was guiding the data analysis was the development of categories by describing their properties and dimensions. When analyzing their interrelationships, a coding paradigm scheme that had been introduced by Strauss and Corbin (33) was applied. In multiple steps, categories were developed that could explain the observed variation in social work practice or reflect the observed and described variation. The researchers elaborated category-based case descriptions (31) for a better understanding of organizational conditions. Additionally, they wrote memos and conducted the entire analysis with support of MAXQDA, a qualitative data analysis software (36). By discussing all hypothesized relationships between those categories with colleagues in workshops, participating social workers and in an annual board meeting, the conclusions were crosschecked and validated intersubjectively. While both social workers and leading staff identified societal expectations in their interviews, this article focuses on the discourse of the social workers, if not labeled otherwise.

## 3 Results

Table 2 provides an overview of the characteristics of the participating rehabilitation facilities and social workers. It is important to note that the study included a high proportion of women and that the average length of professional experience of the participants was relatively long. However, these factors represent the general situation well. The facilities that were recruited are representative of the German rehabilitation system. The staffing ratios of the facilities were found to be above and below the DRV recommendations. Nevertheless, other important characteristics, such as the interprofessional integration of social services, only emerged when the data was analyzed. The researchers conducted interviews with 29 social workers (average duration: 81.3 min), 13 leading staff representatives (42.9 min) and wrote 43 observational protocols [observed situations: e.g., counseling sessions, team meetings, group work and consultation hours (sum of observed hours: 140.3 h)]. The participating social workers recorded 14 counseling sessions (30.5 min).

**Table 2:** Characteristics of the participating rehabilitation facilities and social services.

Rehabilitation facilities		Employees social services	
Variable	k=	Variable	*n*=; Mean
Indication		Sex	
Orthopaedics	6	Male	4
Cardiac/internal medicine	5	Female	25
Psychosomatic	3	Professional experience (in years)	
Oncology	3	Total	14.1
Neurology	4	In rehabilitation	8,4
Other (pneumology, geriatrics)	3	Education in social work	
Rehabilitation service		Diploma	14
Inpatient	2	Bachelor	9
Inpatient & outpatient	6	Master	3
Outpatient	2	No Social Work degree	3
Provider		Other education	
Non-profit	3	Nursing	3
For-profit	7	Administration training	2
Work-related medical rehabilitation^a^		Nursery school teacher	1
Yes	4	Theology	1
Number of beds		Public Health	1
≤200	5	No other education	20
200–400	3		
>400	2		
Employees social service			
2	3		
3–4	5		
≥5	2		
Case load			
1:≤100	7		
1::>100	3		

^a^ *medizinisch-berufliche Rehabilitation (MBOR)*, specialised rehabilitation intervention with both medical and vocational interventions for high risk rehabilitants.

Social workers were confronted with different mandates in the rehabilitation practice and the alignment of these was a significant challenge, as the experienced social worker A summarizes.

*First of all, we do general social counseling […], where we can pick up every possible question that the patients have when they come to us. This is then structured in more detail, when we listen to their concerns. If we look at that and want to classify the problem described by the rehabilitant in this context, we have to consider the mission rehabilitation has, too. (Facility 1/Interview with social worker A: 58–65)*

Some social workers described these challenges and the subsequent (inter-)actions/emotions. In addition, we observed and recorded these phenomena. This enabled the researchers to reconstruct the social work practice when considering the triple mandate. The definition of the mandate as a directive without a concrete call to action is supported by the data. In all three cases, the social workers had to *translate* the mandates into concrete actions.

Firstly, we will present the mandates in isolation, some of the indicators in the data that point to the respective mandate, and the consequences of these mandates to social work practice. Secondly, we will describe the conflicting occurrence of two or more mandates and the subsequent strategies employed by the social workers.

### 3.1 The mandates in social work practice in rehabilitation

The societal mandate has no direct downstream in the social work practice. Some social workers argued with terms like “vetting” or “pension chasers”, but in partial agreement with Staub-Bernasconi the societal mandate was forwarded not by the facilities but by rehabilitation physicians. The main referral to the social services by the physicians explains the significance of this forwarding easily. Such referrals were made by physicians when they identified a need for counseling in their interviews or rounds with patients, as evidenced by the reports of the interviews with the leading physicians or social workers. An assessment of the patientś work (dis)ability often precedes these referrals. The two-dimensional evaluation of the patients’ work ability determines the socio-medical possibilities for their vocational participation. Consequently, the physicians establish parameters for the social law options available to the patients and the social worker. As evidenced by the quote from Social Worker A, they create a background against which the client's concerns must be classified. In essence, this background is defined by two possibilities: either the patient is assessed to have the potential to resume work in his or her original field, or in any other field, or he or she can neither return to his or her previous job nor work in any other area. This evaluation therefore guides the social work practice in the most comprehensive way. As part of the helping dimension of the mandate of the society and because of the evaluation, social workers often inform about and sometimes actively support applications for social benefits by the patients. These activities account for a large amount of work. Social services encounter difficulties when the rehabilitation team does not inform about the evaluation early and in time. Social workers were in almost all participating rehabilitation facilities challenged with the disclosure of the results of the evaluation to the patients and the communication of the consequences of this evaluation for the patients’ vocational perspective. This was due to a lack of effective information management within the rehabilitation team, which resulted in the disclosure being unintentional and sometimes leading to tense situations and necessitating the intervention of social workers.

Some social workers challenge the physician's recommendations. One possible explanation for this is that they believe that the rehabilitation physicians do not or cannot sufficiently consider the patients’ contextual factors that account for the client's mandate. This is also expressed by the fact that the social workers sometimes receive insufficient information about the reasons for referral by the physician. As a result, the social workers often describe the first encounter after a referral as a situation where one never knows what to expect. In order to address this openness, the social services apply some strategies in the preparation of the encounters. This consideration by the social workers is a first indicator of a strong emphasis towards the autonomy of the clients and their mandate. Other indicators are the underlining of the significance of consultation hours and the highlighted provision of time to address self-initiated concerns in groups. The social workers associated both of these strategies with the goal of securing equity. Some social workers express this strict patient-oriented approach by stating the primacy of this mandate when talking about their practice.

*So, the patient*’*s wish or the patient*’*s idea is still the main guideline. When they say: „But I would like to try to go to work“, even if this is absolutely not advisable for medical reasons, then I would accept/ (.) it is just like that. Their words are the law. Then we would just hand out the application form for vocational rehabilitation and explain it to them [, instead of filing the application form and applying for it] (Facility 7/Interview with social worker B: 385–392)*

Social Worker B uses vocabulary reminiscent of the discourse around the support in decision-making based on an examination of current evidence (i.e., “guideline”). She identifies the clients’ views as normative (i.e., “their words are the law”) as a counterweight to a societal mandate. Her wording is remarkable and it appears to reflect the consequences of this attitude for the professional practice. The quote from Social Worker B is in agreement with the people-first principle of Staub-Bernasconi (23), but in contrast to other social workers. For instance, Social Worker C challenges the clients’ mandate in the following quote and encourages a critical reflection concerning the patient's health.

*Rehabilitant R1: My husband earns 450 euros (C: M-hmm.), I cannot consider my health all the time. I have to consider what*’*s good for both of us.*

*Social worker C: Yees, what should I say? I would always say “health first”, for sure, but I can relate to your dilemma of course. But (R1: But, if/) maybe in the long run, you won*’*t get anything out of it as a couple, if you then are completely exhausted at some point. Then it doesn`t get any better. (Facility 6\counseling session of social worker C: 504–513)*

Social workers with an emphasis on the clients’ mandate tend to approach a more co-productive practice and speak up for the clients’ concerns in team meetings. Co-production describes the extent to which social workers hand over the responsibility for action to the patients. The continuum extends in almost all identified work types from the complete handover to a complete takeover of these actions. The advocacy social workers demonstrated in team meetings had consequences for their standing in the rehabilitation team. Social workers with a strong emphasis on the clients’ mandate fulfilled the role of advocates for the rehabilitants in interprofessional team meetings. In interviews, some of the leading medical practitioners described them as overly caring, patronizing or overly dedicated. Other leading staff members underscored the commitment of the social workers.

Another possible explanation for the importance of the clients’ mandate in the data is the patients’ often limited ability to articulate a mandate. In such instances, social workers would have spent time in identifying the client's interests and assumed an advocacy function. However, the professional obligation does not include this, as Social Worker D criticized when describing the aforementioned preparation of first encounters.

*If there are five, six, seven other concerns [social worker D hypothesizes] and „Initiate aftercare“ is my task with the patient, I can easily assume that this is really not the end of the story. If I did what I was told to do and if the patient does not come up with other concerns him or herself, my job is done here. If there is nothing, there is nothing. […] But there isn*‘*t just nothing. Maybe they don*’*t dare or because I say it*’*s about aftercare today they don`t say anything […] In the end, the question for me is always: “Are those people in good hands when coming home again?” (Facility 10/Interview with social worker D: 100–121)*

While observing her practice, Social Worker D explains further that there are no professional standards guiding her practice. She states that there would be no consequence for her actions even if they were substandard, given the lack of awareness of the actual scope of social services (Facility 10/protocol of participant observation I by TK: 547–550).

The first quote from Social Worker A did not report a professional reflection of the mandates, which is the core of the third mandate within the profession. Social Worker B, who orientates herself solely to the client's mandate, did not engage in a similar reflection. However, social workers did contemplate the other mandates and, on occasion, issued mandates independently.

*After the rehabilitant*’*s (R2) further perspective has been clarified, Social Worker E asks whether R2 has already thought about how he would like to say goodbye to his company. She notes that she thinks it would be important for him to have a good farewell. R2 agrees with her. (Facility 3/protocol of participant observation I by TK: 348–352)*

In addition to an in-depth or repeated training and coordination of the care transition, these mandates often relate to supportive activities in the development of a participation perspective. These were, for instance, the personal development as Social Worker E addressed in the situation above or the raising of and promoting resources. When reflecting on the other mandates, social workers expressed arguments that were more grounded in their professional experience and moral implications than in theoretical or research-informed considerations.

*If a patient sits in my office and I have a bad feeling about the suggestion made by the medical practitioner. Then I always try to get feedback from the psychotherapist and then check again whether I want to talk to the medical practitioner again. Ultimately, it is their decision. […] Some [medical practitioners] can change their minds and say “Okay.“ and others just state “No, I see it that way.” (Facility 3/Interview with Social Worker F: 915–944)*

### 3.2 Conflicting mandates and subsequent strategies by social workers

So far, this paper has only discussed the social workerś consecutive strategies when emphasizing one mandate. However, as indicated by the explanation of the appearance of the professional mandate in the data and the alignment described by Social Worker A, conflicts can occur when mandates are incongruent. Such conflicts were expected *a priori* by the researchers and were briefly discussed in the section on the first mandate. Subsequent strategies from these situations are the focus of the following section. The first strategy explained is *empathy and objectivity*.

This category summarizes the social work activities that integrate the conflicting mandates. On the one hand, social workers possess a comprehensive knowledge of social law and educate the clients about their eligibility for welfare benefits. On the other hand, these activities are carried out in the context of the client ’s individual situation and with a degree of empathy that leads to taking the patient's concerns seriously. A case in point is the observed counseling session between Social Worker A and the nearly 60-year-old, blue-collar patient 3 (R3). In this instance, the social worker demonstrates an awareness of the legal framework while prioritizing the patient's needs.

*R3 says that he would like to apply for a disability pension. Social Worker A does not think that he has a good chance of being approved. She states that he does not fulfill the medical requirements and she refers to the positive work-ability evaluation. In the course of the interview R3 and A talk about R3*’*s work. He works as a warehouse worker. It becomes clear that the physical job requirements cause fewer problems than the progressive computerization of his job. A speculates that his desire for the disability pension stems from excessive demands which is confirmed to her by R3. She explains that this, however, is not a reason for a disability pension and instead points out other options such as vocational rehabilitation. (Facility 1/Protocol of participant observation I by NA: 424–430)*

Another strategy reflects the interrelation between the mandates of professionals and clients. In paternalistic approaches, social workers overrule the client's mandate by deciding in in what he/she assumes to be beneficial to the patient. As Social Worker F reported in the quote above, some social workers try to build alliances with other professionals to challenge recommendations of the medical practitioner. Important preconditions reconstructed from the data are the possibilities of interprofessional collaboration and the position of the social workers in the rehabilitation team. Consequently, not all social workers had the opportunity to build alliances. Maybe because of this, but also independent of this condition, the social workers did indeed confront the medical practitioners with the clients’ concerns. For instance, Social Worker G called the leading physician to discuss her divergent views of the patients’ work capacity. With this action, Social Worker G made a break in his routines, as he told the observer of this situation, as he had never done anything like this before (Facility 1/protocol of participant observation I by TK: 215–21). Another strategy, which is diametrically opposed to the aforementioned approach, is when social workers do not consider the clients’ concerns. We could identify numerous instances of these strategies and categorize them as a retreat to the law. When confronted with the client's displeasure with the assessment, the social workers simply refer collectively to social law without addressing individual claims or, if still necessary, they refer to the medical practitioner for further discussion.

## 4 Discussion

In this paper, we apply the triple mandate as a theoretical lens to analyze data on vocational participation as an important issue in rehabilitation. In the analysis, the social services focused on the societal or client mandate. The physicians forwarded the mandate from society. These orientations had direct consequences for social work practice, as the mandate from society often limits the possibilities under social law. Social workers often drew on their professional experience when reflecting on the mandates. They used a variety of strategies when faced with conflicting mandates. Only one strategy, empathy and objectivity, had an integrative approach. The other strategies reflected an emphasis on the client's point of view or the demands of society.

To conclude the data analysis, the mandates recommended by Staub-Bernasconi (22) do not cover all the requirements. The forwarding of the social mandate by the medical practitioner is, strictly speaking, a theoretical artifice. Consequently, it is necessary to ascertain whether medical practitioner experience the same conflicts of loyalty as social work theory would suggest, and whether their conflicts of loyalty are identical to those of the social workers in this sample. If this is the case, the forwarded mandate is more than the mandate from society; it is an already ethically processed outcome between the professional identity of the medical practitioner, perhaps the views of the medical practitioner and the demands of society (37). Further research into the action/interaction of medical practitioner with these tasks would lead to a deeper understanding of this social work mandate. As rehabilitation has also an interprofessional approach, this multidimensional consideration of ethical issues is not exclusive to social work. We have already shown that other rehabilitation professionals are also challenged. Rehabilitation research has addressed the ethical issues of other professionals, including physiotherapy (28), occupational therapy (20), and the entire rehabilitation team (20). Further research should explore the potential of the theoretical lens applied here to address ethical issues related to the whole rehabilitation team.

### 4.1 Limitations and strengths

The ten rehabilitation facilities we have included in the study reflect the main patient groups and types of rehabilitation. However, in terms of a stochastic understanding of representativeness, this sample could not be considered representative. The SWIMMER project's qualitative sampling strategy was guided by maximum variation sampling (32) using criteria relevant to variation in social work practice and “representativeness of concepts” (33). The first aim was fully met, while the second was only partially met. Prior to this research project, the only reference points for explaining variation were the diagnostic groups, the type of rehabilitation service and the presence of the necessary resources for so-called work-related medical rehabilitation. Table 2 shows that all three variables were sufficiently included. After collecting data in the first six facilities, the researchers used a break in data collection to conduct a preliminary data analysis and to develop initial hypotheses to guide the subsequent theoretical sampling. Not all of the criteria developed could be met with the inclusion of the last four participating clinics. As a result, not all possible variations of the initial hypothesis could be tested or further developed. The original intention was to compare a specific analyzed facility with a similar institution in the sense of a minimal contrast. To achieve this, we tried to include an outpatient rehabilitation facility in a large city with a specialization in psychosomatic rehabilitation. Due to the lack of access to such a facility, the hypothesized importance of a community-based approach in this facility could not be further evaluated or tested with new data. We hypothesized that this approach ensures direct contact with employers, which can be an important asset in dealing with goal conflicts in vocational participation.

The data collected allowed us to make assumptions about the possible influence of the triple mandate on social work practice. However, the extensive data collection and efforts at intersubjective validation, as well as the case-contrasting approach, make these assumptions empirically grounded and robust. Future studies should operationalize the categories developed and confirm these assumptions in a standardized research approach.

As a theoretical point of departure, we selected the mandate theory of Staub-Bernasconi (20), which allowed us to understand the (goal) conflicts experienced by social workers as ethical conflicts of different values. This approach proved to be more appropriate to the object of interest than other widely-used ethical frameworks, such as the ethical principle of Beauchamp and Childress (19).

The focus on vocational issues excludes a significant part of social work practice, especially in neurology and oncology, where the main focus of rehabilitation is not return to work, but social participation or health-related. The triple mandate is also present in these cases, as rehabilitants do not want to go to a specific nursing home or discuss treatment regimens. It is also important to investigate these settings further, as emotions such as compassion may have a potential impact in the oncology setting, for example.

We must also acknowledge that the restrictions due to the COVID-19 pandemic changed rehabilitation practice. As a result, many social work interventions could not be observed due to restrictions on visiting rehabilitation facilities, which is a limitation of our study. However, in the interviews the social workers often compared their practice to the time before the COVID-19 pandemic. These comparisons were often helpful in the analysis. The restrictions resulted in an underrepresentation of social workers’ personal contacts with employers, family, friends, or external professionals. Given the potential role these actors can play in resolving or exacerbating conflicts, a significant amount of social work practice was not accessible. Despite the fact that employers were often passive recipients of information, the consideration of patients’ social networks was not prominent in the social workers’ daily routines.

The focus on professionals’ actions is a strength of this research. Most previous studies have used a descriptive and quantitative approach. In this study, however, it was possible to analyze the ethical issues and their consequences for action, as McGlinchey & Davenport did (28). In their definition of rehabilitation, Negrini et al. (13) highlight the importance of a person-centered approach that considers the values, preferences, and contextual factors of the individual in question. However, this research takes a broader view and combines the perspective of the rehabilitee with a professional view and the demands of society.

All interviews were conducted in German. For the purposes of this publication, quotes have been translated. It is important to note that this translation may not convey important nuances within the quotes.

### 4.2 Further implications for research and practice

We have described the potential impact of mandates on social work practice, but we have not paid attention to the “ethics work” (38) of social services. Staub-Bernasconi's contribution to the management of potential conflicts is also limited. Social workers told us that much of their work is invisible. The ethical work they do is also invisible, but they work in a morally charged environment. Given the aim of this paper, further research into the emergence of the different emphases identified is an important next step. We can only guess at some of the factors relevant to the development of a particular focus within the triple mandate. These are professional experience, education and training and the professional identity of social workers. To follow up on this question, one could use the operationalized categories mentioned above and survey social workers’ attitudes toward the triple mandate and the factors mentioned. This research should be accompanied by a more descriptive approach and identify other relevant ethical issues.

The findings of this paper also have some implications for social work practice in rehabilitation. This research provides evidence of where cultural dependencies (39), particularly on the social (welfare) system and its regulations, influence social work practice in rehabilitation. The research design does not allow for a comparison of social services in terms of effectiveness, but the integrative strategy, for example, highlighted the particular skills of social work in the rehabilitation team and can be linked to some promising cases in the data. However, this strategy is more complex than just *empathy and objectivity* and therefore takes more time. Social work education in rehabilitation should therefore focus on counseling and clinical reasoning skills to elicit the client's mandate, as well as legal education, i.e., the competence to apply knowledge of social law to the client's individual situation. Although this is not a direct finding on the triple mandate, a closer look at the mandate of clients, who often find it difficult to articulate their mandate, and the conditions of referral to social services make an investigation of assessments seem advisable. Staub-Bernasconi promotes the professionalization of social work through the formulation of a third mandate (23). It is important to note that our findings are consistent with those of James et al. (40), who also observed a lack of evidence-based and theory-driven thinking in social work practice. However, the third mandate is not sufficiently defined. Acting ethically and based on the best evidence is reminiscent of the evidence-based rehabilitation framework (cf. 41). Despite the challenges that social work may face in implementing this approach (11), the results of our study underscore its necessity. It is therefore essential to make the necessary ethical considerations or ethics work (38) more explicit and to conduct further evaluations of social work interventions. Staub-Bernasconi states that the third mandate is a prerequisite for the professional independence of social work in medical rehabilitation, i.e., not being bound by instructions (22). As social workers in rehabilitation are part of a team with a case-responsible physician or psychotherapist (3), the objective is not professional independence but rather to become an established member of the rehabilitation team with a clearly delineated area of responsibility. The structural dominance of medicine in this field and the perceived informational dependence of social workers on physicians in our data challenge this mandate. The valuation of interprofessional teams and the established presence of social workers in this team may be considered potential prerequisites for the articulation of a sound professional mandate, which is evidence- and theory based, as well as ethically underpinned.

## Data Availability

The datasets presented in this article are not readily available because anonymity protection. Requests to access the datasets should be directed to Tobias Knoop, tobias.knoop@uk-halle.de.
