# Corrigendum: On the development of a professional mandate by social workers in medical rehabilitation- key results from the SWIMMER Project

**DOI:** 10.3389/fresc.2024.1508335

**Published:** 2024-10-30

**Authors:** Tobias Knoop, Nadja Freymüller, Stephan Dettmers, Thorsten Meyer-Feil

**Affiliations:** ^1^ Faculty of Medicine, Institute for Rehabilitation Medicine, Interdisciplinary Centre of Health Sciences, Martin Luther University Halle-Wittenberg, Halle (Saale), Germany; ^2^ Endowed Professorship Rehabilitation Science | Health Services Research in Rehabilitation, School of Public Health, Bielefeld University, Bielefeld, Germany; ^3^ Institute for Social Work in the Life Course, OST-Ostschweizer Fachhochschule, St. Gallen, Switzerland

**Keywords:** goal orientation, professionalism, health services research, ethnography, social law, interprofessional collaboration

A Corrigendum on On the development of a professional mandate by social workers in medical rehabilitation- key results from the SWIMMER project By Knoop T, Freymüller N, Dettmers S, Meyer-Feil T (2024). Front. Rehabil. Sci. 5:1383995. doi:10.3389/fresc.2024.1383995

**Error in the Article Title**

In the published article, there was an error in the article title. Instead of “One the development of a professional mandate by social workers in medical rehabilitation– key results from the SWIMMER Project”, it should be “On the development of a professional mandate by social workers in medical rehabilitation– key results from the SWIMMER Project”.

The authors apologize for this error and state that this does not change the scientific conclusions of the article in any way. The original article has been updated.

